# Acclimation of Foliar Respiration and Photosynthesis in Response to Experimental Warming in a Temperate Steppe in Northern China

**DOI:** 10.1371/journal.pone.0056482

**Published:** 2013-02-15

**Authors:** Yonggang Chi, Ming Xu, Ruichang Shen, Qingpeng Yang, Bingru Huang, Shiqiang Wan

**Affiliations:** 1 Key Laboratory of Ecosystem Network Observation and Modeling, Institute of Geographic Sciences and Natural Resources Research, Chinese Academy of Sciences, Beijing, China; 2 University of Chinese Academy of Sciences, Beijing, China; 3 Department of Ecology, Evolution and Natural Resources, Center for Remote Sensing and Spatial Analysis, Rutgers University, New Brunswick, New Jersey, United States of America; 4 Huitong Experimental Station of Forest Ecology, State Key Laboratory of Forest and Soil Ecology, Institute of Applied Ecology, Chinese Academy of Sciences, Shenyang, China; 5 Department of Plant Biology and Pathology, Rutgers University, New Brunswick, New Jersey, United States of America; 6 Key Laboratory of Plant Stress Biology, College of Life Sciences, Henan University, Kaifeng, Henan, China; The Ohio State University, United States of America

## Abstract

**Background:**

Thermal acclimation of foliar respiration and photosynthesis is critical for projection of changes in carbon exchange of terrestrial ecosystems under global warming.

**Methodology/Principal Findings:**

A field manipulative experiment was conducted to elevate foliar temperature (*T*_leaf_) by 2.07°C in a temperate steppe in northern China. *R*_d_/*T*_leaf_ curves (responses of dark respiration to *T*_leaf_), *A*_n_/*T*_leaf_ curves (responses of light-saturated net CO_2_ assimilation rates to *T*_leaf_), responses of biochemical limitations and diffusion limitations in gross CO_2_ assimilation rates (*A*_g_) to *T*_leaf_, and foliar nitrogen (N) concentration in *Stipa krylovii* Roshev. were measured in 2010 (a dry year) and 2011 (a wet year). Significant thermal acclimation of *R*_d_ to 6-year experimental warming was found. However, *A*_n_ had a limited ability to acclimate to a warmer climate regime. Thermal acclimation of *R*_d_ was associated with not only the direct effects of warming, but also the changes in foliar N concentration induced by warming.

**Conclusions/Significance:**

Warming decreased the temperature sensitivity (*Q*_10_) of the response of *R*_d_/*A*_g_ ratio to *T*_leaf_. Our findings may have important implications for improving ecosystem models in simulating carbon cycles and advancing understanding on the interactions between climate change and ecosystem functions.

## Introduction

The balance between respiration and photosynthesis is critical to the exchange of carbon between the atmosphere and the terrestrial biosphere [1]–[3]. Instantaneous increases in foliar temperature (*T*_leaf_) typically result in an increase in respiration/photosynthesis (*R*/*A*) ratio because the response of respiration to *T*_leaf_ normally follows an approximate exponential-type curve (at moderate temperatures) while the response of photosynthesis to *T*_leaf_ often bears a bell-shaped curve [i.e. the thermal optimum (*T*_opt_) of respiration is higher than that of photosynthesis] [4], [5]. In contrast, long-term warming experiments have suggested that *R*/*A* ratio is often conservative to changes in growth temperature (*T*_growth_) through acclimation, the metabolic adjustment for compensating changes in *T*_growth_ [6]–[8]. Acclimation could occur via suppression of respiration in response to changes in foliar carbohydrate supplies [4], [9]. The thermal acclimation of respiration and photosynthesis is associated with multitudes of signal cascades and networks, which involves the reallocation of resources to achieve and maintain not only optimal *R*/*A* ratio but also protective strategies under sustained warming as projected by global climate models [10]–[12]. However, the mechanisms of thermal acclimation of respiration and photosynthesis to climate warming are far from clear, especially in natural ecosystems.

The acclimation of foliar respiration to warmer *T*_growth_ has been found in numerous studies [8], [13]–[17], which may also be associated with plant developmental stage and other abiotic factors, such as drought and nutrient availability [18]–[21]. Thermal acclimation of respiration might occur via changes in the temperature sensitivity, *Q*_10_, or the basal respiration, *R*_10_ (respiration at a reference temperature, such as 10°C) [11]. Altered *Q*_10_ partially reflects temperature-mediated changes in energy demand and/or available substrates [1], [17], [20] whereas changes in *R*_10_ may be associated with temperature-mediated changes in respiratory capacity, reflecting changes in mitochondrial abundance, structure and/or protein composition [22]–[24]. As a result, thermal acclimation of respiration may enhance plant net carbon assimilation by reducing carbon loss under warmer *T*_growth_ while maintaining basal rates of respiration in colder *T*_growth_ for subsequent recovery [12], [20], [25], [26].

The thermal acclimation of the foliar net CO_2_ assimilation rate (*A*_n_) may involve three primary sets of processes that control the *A*_n_/*T*_leaf_ curves (response of *A*_n_ versus *T*_leaf_), namely respiratory, biochemical and stomatal processes [27]. First, *A*_n_ is the difference between gross CO_2_ assimilation rate (*A*_g_) and foliar dark respiration (*R*_d_), *A*_n_ = *A*_g_ – *R*_d_, which requires the decoupling of the two processes because *A*_g_ and *R*_d_ feature different thermal dynamic properties and thus involve different thermal acclimation processes [28]. This could result in a shift in *T*_opt_ and a change in the shape of the *A*_n_/*T*_leaf_ curve. Therefore, *R*_d_ must be evaluated separately and factored out to understand the acclimation mechanisms of *A*_g_ in response to global warming [3], [18], [29]. Second, the acclimation of *A*_g_ to warmer *T*_growth_ deals with the changes in Rubisco activity [29]–[33] and electronic transport processes [34] where *T*_growth_ affects the thermal dependence of various enzymes in the dark and light reactions [35], [36]. Therefore, the temperature sensitivity of the maximum rate of Rubisco carboxylation (*V*_cmax_) and the maximum rate of photosynthetic electron transport (*J*_max_) are associated with the acclimation of *A*_g_ [36], [37]. In addition, the change in the balance between carboxylation and regeneration of RuBP, indicated by *J*_max_/*V*_cmax_ ratio, may also result in the shift of *T*_opt_ of *A*_g_ due to nitrogen (N) partitioning in the photosynthetic apparatus [3], [31], [38], [39]. Finally, the temperature-dependent diffusion processes of CO_2_ to chloroplasts, such as stomatal conductance (*g*_s_) and mesophyll conductance (*g*_m_), can also affect the thermal acclimation of photosynthesis [36], [40]. Kirschbaum and Farquhar [41] showed that higher conductance could cause an increase of CO_2_ concentrations in the carboxylation site (*C*_c_) and then resulted in a shift in limitation of *A*_g_ from Rubisco to electron transport capacity. Since *T*_opt_ of electron transport-limited *A*_g_ is higher than that of Rubisco-limited *A*_g_, *T*_opt_ of *A*_g_ was increased (0.05°C per 1 µmol mol^−1^ CO_2_) [36].

*Stipa krylovii* Roshev. is a keystone species in the temperate steppe in northern China [42], [43]. Climate models predict this region will be 4°C warmer by 2100, which may have severe impacts on *Stipa krylovii* Roshev. [44]. Examining the respiration and photosynthesis of this species is critical to the steppe productivity and the carbon cycle of the ecosystem. The objectives of the current study are to examine: (1) the acclimation capacity of respiration and photosynthesis to experimental warming under field conditions, and (2) the homeostasis of respiration/photosynthesis ratio in response to experimental warming in the steppe ecosystem.

## Materials and Methods

### Site Description

The research site (42°02′ N, 116°17′ E, 1324 m a.s.l.) is a typical temperate steppe located in Duolun County, Inner Mongolia Autonomous Region, China. The experiment has received the permits for the field study from the land owner, Institution of Botany, Chinese Academy of Sciences. The mean annual temperature (MAT) is 2.1°C, with monthly mean temperature ranging from −17.5°C in January to 18.9°C in July. The mean annual precipitation (MAP) is approximately 385 mm with approximately 85% falling from May to September. The soils are chestnut (Chinese classification system) or Haplic Calcisols (FAO classification system), with 62.8% sand, 20.3% silt, and 17.0% clay respectively. The soils are characterized as sandy, slightly alkaline and nutrient poor with pH values around 7.7 and bulk density of 1.3 g cm^−3^ and soil total organic C and N concentrations of 16.1 and 1.5 g kg^−1^ respectively. The plant communities in the temperate steppe are dominated by *Stipa krylovii* Roshev., *Artemisia frigid* Willd., *Potentilla acaulis* L., *Cleistogenes squarrosa* (Trin.) Keng., *Allium bidentatum* Fisch. ex Prokh., and *Agropyroncristatum* (L.) Gaertn.

### Warming Experiment

**Figure 1:**
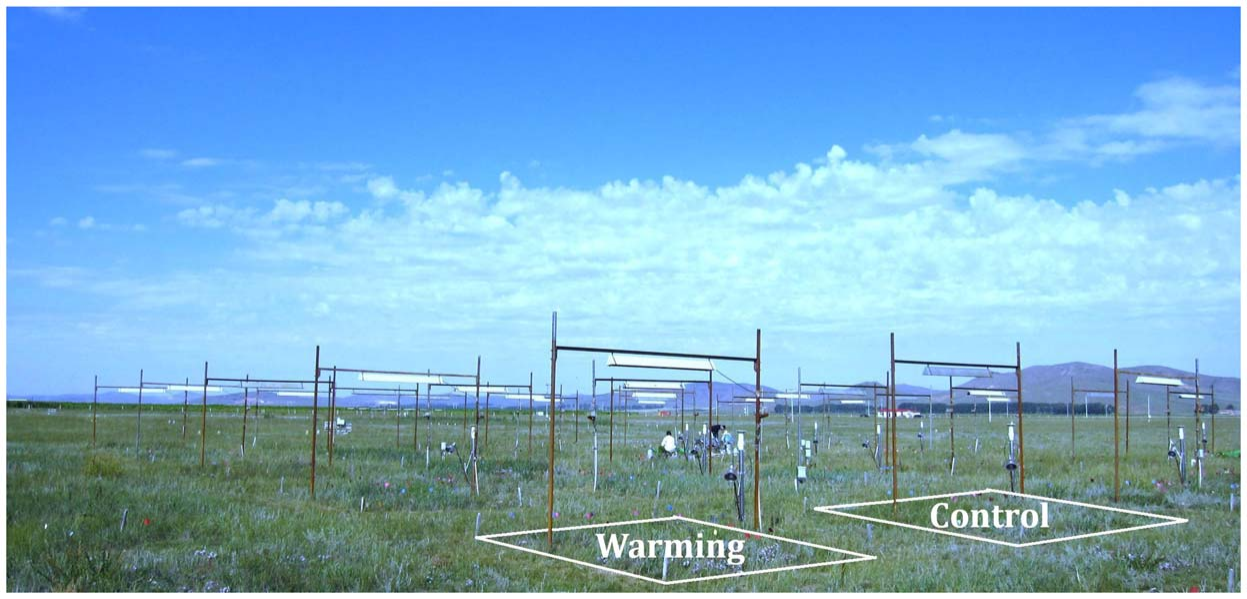
Layout of the experiment plots in a temperate steppe in northern China. Infrared heaters were suspended as the heating sources at the warming plots while ‘dummy’ heaters were suspended to simulate shading effects of the infrared heater at the control plots.

The warming experiment was initiated in April 2006 with infrared heaters (MSR-2420, Kalglo Electronics Inc., USA; radiation output is approximately 1600 W) as the heating source (Fig. 1). Briefly, an infrared heater of 1.65 m in length was suspended at 2.25 m above the ground in each warming plot which features a dimension of 3×4 m. A reflector associated with the heater can be adjusted so as to generate an evenly distributed radiant input to the plant canopy. In the control plots, a ‘dummy’ heater with the same shape and size was suspended at the same height to simulate shading effects of the infrared radiator. The effects of warming on *T*_leaf_ were measured using a portable infrared thermometer (FLUKE 574, Fluke Inc., USA). The mean daytime *T*_leaf_ in the warming plots was increased by 2.07°C compared to the control plots. The warming experiment was designed for long-term simulation of global change and it featured a complete random block design with multiple treatments (day warming, night warming, diel warming, and N addition) and six replicates. We took advantage of this multi-factor experiment by selecting the diel warming and control plots with all the other factors kept at control levels. The details of the experiment can be found in Wan *et al*. [44] and Xia *et al*. [45].

### Gas Exchange Measurements

**Figure 2:**
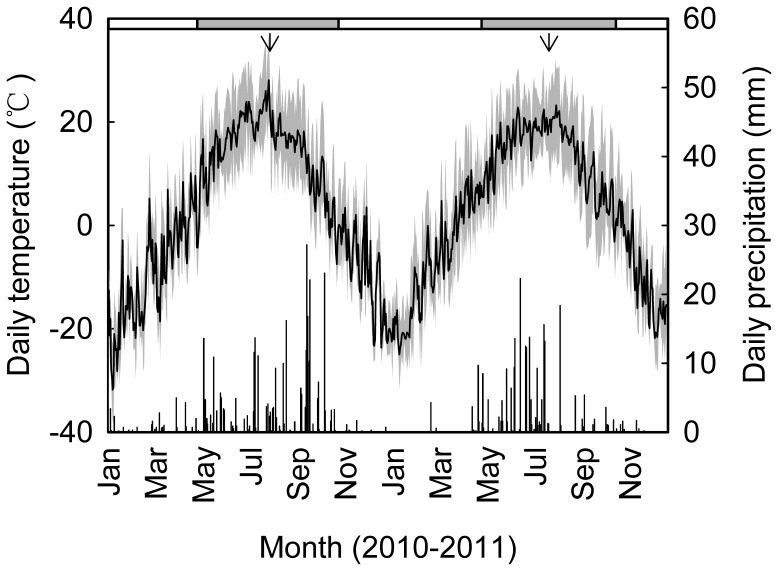
Daily maximum, minimum and mean air temperature (lines) and precipitation (bars) at the study site in 2010 and 2011. The filled rectangles on the top of figure indicate the growing season (May to October) and the open rectangles for the non-growing season (November to April). The arrows mark the timing of field campaigns when the gas exchange measurements were initiated.

We measured foliar gas exchange using a portable photosynthesis system (LI-6400, LI-COR Inc., USA) in the middle of the growing seasons (late July to early August) in 2010 and 2011 (Fig. 2) to remove the effect of seasonal changes in photosynthetic and respiratory acclimation in *Stipa krylovii* Roshev. [19]. Four individuals (one individual per plot) were measured in each treatment. Eight days were required to complete all field measurements each year. Light, *T*_leaf_, humidity, and CO_2_ concentration were independently controlled in a 2×3 cm cuvette. Given the *T*_leaf_ control capacity is limited (within ±6°C) with the factory setup of the LI-6400 system, we modified the *T*_leaf_ control system by adding metal blocks with water channels to heat or cool the peltiers, thermoelectric cooling elements. The water channels were connected to a heating/cooling water bath whose temperature was controlled by adding hot water or ice. This modification allows holding *T*_leaf_ at any level between 10 and 40°C during the summer growing season in the steppe.

The photosynthetically active photon flux density (PPFD) was provided by the red/blue LED light source built in the foliar cuvette calibrated against an internal photodiode (LI-6400-02B, LI-COR Inc.). The vapor pressure deficit (VPD) in the foliar cuvette was controlled by passing the air entering the cuvette through either anhydrous calcium sulfate for the lower *T*_leaf_ when humidity was high or bubbling air via water at higher *T*_leaf_ when the air was dry. CO_2_ concentrations in the cuvette were controlled using an injector system (LI-6400-01, LI-COR Inc.) which functions with a CO_2_ mixer and compressed CO_2_ cartridges. Cuvette was sealed with plasticine to prevent leakage. Potential leakage of CO_2_ out and into the empty cuvette was determined for each concentration and used to correct the measured foliar fluxes with the equations provided by von Caemmerer and Farquhar [46] and Galmés *et al*. [47]. The gas exchange system was zeroed using H_2_O and CO_2_ free air every day.

Typical *A*_n_/*C*_i_ curves (*A*_n_ versus calculated intercellular CO_2_ concentrations, *C*_i_) were measured at *T*_leaf_ changing from 10 to 40°C with 5°C increments each. We started with the *A*_n_/*C*_i_ curves at low *T*_leaf_ (10°C) in the morning around 7∶00 am finished at high *T*_leaf_ around noon. As to the problem of co-variance between the daily cycle and temperature, Luo *et al.* [48] and Way and Sage [3] suggested that the observed responses in the biochemical parameters resulted mainly from changes in temperature rather than changes in time of day. It usually took *c.* 5 min for *T*_leaf_ to reach stability at each step change in temperature. Photosynthesis was induced for 10 min in saturating PPFD (1500 µmol photons m^−2^ s^−1^) and at ambient CO_2_ concentration (*C*_a_) of 380 ppmv. Measurements were made at saturating light (1500 µmol photons m^−2^ s^−1^), and a leaf VPD between 0.5 and 2.0 kPa, except for 40°C where the VPD was 4.5±0.05 kPa. *A*_n_ was measured at cuvette CO_2_ partial pressures between 50 and 1200 ppmv CO_2_. The *C*_a_ was lowered stepwise from 380 to 50 ppmv and then increased again from 380 to 1200 ppmv with the total of 9 points. In total, 112 *A*_n_/*C*_i_ curves were measured and used for the analysis of physiological parameters in this study. *A*_n_/*T*_leaf_ curves (response of light-saturated *A*_n_ at 380 ppmv versus *T*_leaf_) were obtained based on the *A*_n_/*C*_i_ curves measured from 10 to 40°C.

*R*_d_ was measured by turning off the LED light source for at least 5 minutes in the cuvette after each *A*_n_/*C*_i_ curve was accomplished [49]. All other conditions were the same as *A*_n_/*C*_i_ curve measurements. Measurements of *R*_d_ on previously illuminated leaves were performed after a period of darkness in order to avoid light-enhanced dark respiration (LEDR) [13], [18]. Five data points of *R*_d_ were logged at a 30 s interval and averaged for *R*_d_ at a given *T*_leaf_. *A*_g_ was calculated by adding *R*_d_ to *A*_n_ at each *T*_leaf_.

### Estimation of *V_cmax_*, *J_max_*, *TPU* and *g*_m_

*A*_n_/*C*_c_ curves (*A*_n_ versus chloroplastic CO_2_ concentration) were fitted to estimate *V*_cmax_, *J*_max_, *TPU* (triose-phosphate utilization) and *g*_m_. The spreadsheet-based software of Sharkey *et al*. [50] was modified (Appendix S1) to fit the *A*_n_/*C*_c_ curve by fixing the *R*_d_ value which was measured following the *A*_n_/*C*_i_ curve. This modification will improve the model performance by reducing the number of estimated parameters and thus decreasing the degree of freedom in fitting the model. As in the original software the optimum of *V*_cmax_, *J*_max_, *TPU* and *g*_m_ was obtained by minimizing the root mean square error (RMSE) of each curve [51], [52].

### Estimation of Dependence of Reaction Rates on Temperature

The responses of *R*_d_ and *V*_cmax_ to *T*_leaf_ were fitted to a non-peaked model, following Harley *et al*. [53], due to the fact that the deactivation of *R*_d_ and *V*_cmax_ was not observed in our study:
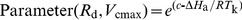
(1)where *c* is a scaling constant, Δ*H*_a_ is the activation energy, *R* is the molar gas constant (0.008314 kJ K^−1^ mol^−1^) and *T*_k_ is the absolute *T*_leaf_ (K) [54]. *Q*_10_ of *R*_d_ and *V*_cmax_ were modeled using the following general function:
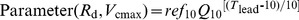
(2)where *ref*_10_ is the estimated basal rate at the reference temperature of 10°C, and *T*_leaf_ is the leaf temperature (°C). The responses of *A*_n_, *A*_g_ and *J*_max_ to *T*_leaf_ were fitted using a peak model in view that the deactivation at high *T*_leaf_ was substantial:
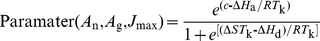
(3)where Δ*H*_d_ is a term for deactivation and Δ*S* is an entropy term [54], [55]. The second derivative of Eqn 3 shows that Topt can be calculated [56] as follows if the parameter includes a peak:
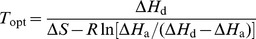
(4)


### Estimation of Biochemical Limitations to Photosynthesis

Temperature dependence of *A*_g_ limited by RuBP carboxylation (*A*_c_), RuBP regeneration (*A*_j_) and *TPU* (*A*_p_) were reconstructed as follows:
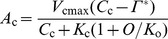
(5)

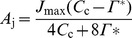
(6)

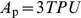
(7)where *V*_cmax_, *J*_max_ and *TPU* were derived from fitted kinetic parameters (*c*, Δ*H*_a_, Δ*H*_d_ and Δ*S*) in our study, *K*_c_, *K*_o_ and *?* ^?^ were derived from a general set of kinetic parameters in Sharkey *et al*. [50]. *C*_c_ was set at 250.8 ppmv in view that the mean *C*_c_/*C*_a_ ratio was 0.66 at ambient CO_2_ concentration (380 ppmv) for all the *A*_n_/*C*_i_ curves measured in the current study, *O* was the partial pressure of oxygen at Rubisco.

### Foliar Characteristics

Foliar N concentration on an area basis was determined using the foliage covered in the cuvette during the gas exchange measurements. The foliage samples were first used to measure the leaf area with an area meter (Li-3100, Li-Cor Inc.) and then biomass where the samples were dried at 65°C for 48 h. Then the dry samples were ground to powder for measuring the total C and N concentrations with a CN analyzer (NA Series 2, CE Inc., Germany).

### Data Analyses

The raw data from the gas exchange measurements were cleaned and processed in Excel spreadsheets where the non-linear *A*_n_/*C*_c_ curve fitting was performed as in Sharkey *et al*. [50]. The fitting was improved by fixing *R*_d_ with the measured value (Appendix S1). Further statistical analyses were conducted using SPSS (version 17.0, SPSS Inc., USA). One-way ANOVA was used to analyze the effects of warming on (1) the foliar chemical properties (C, N, and C/N ratio) and (2) the thermal dynamic properties (*c*, Δ*H*_a_, Δ*H*_d_, Δ*S*, *Q*_10_, *T*_opt_ and *ref*_10_) of foliar gas exchange (*A*_n_, *R*_d_ and *A*_g_) and photosynthetic metabolism (*V*_cmax_ and *J*_max_ ). Differences were considered statistically significant at *P*<0.05. Linear regression was employed to examine relationships between foliar properties and climate (i.e. *T*_growth_). *T*_growth_ in the control plots was an average for daytime *T*_air_ during the 5 d prior to gas exchange measurements in each plot. This choice was based on: (1) our observation that the bulk of individual foliar development by *Stipa krylovii* Roshev. species typically required 4–6 d; and (2) published results indicating that adjustments of foliar metabolism to climate change can occur rapidly (e.g. in a span of 1–5 d following a shift in *T*_growth_ [13], [15], [57]–[61]); (3) Gunderson *et al*. [60] found that *T*_opt_ for photosynthesis was strongly correlated with mean daytime *T*_air_. In addition, *T*_growth_ in the warming plots were approximatively calculated by adding warming effects (2.07°C) to the mean daytime *T*_air_ during the 5 d prior to gas exchange measurements in each plot.

## Results

### Microclimate and Experimental Warming

The meteorological data collected at the experimental site showed that the growing season of 2010 was dry while the growing season of 2011 was wet (Fig. 2). The daily mean *T*_air_ between 1 May, the onset of plant growth, and the time of the field measurements (27 July in 2010 and 2011) was 17.2°C in 2010 and 15.6°C in 2011 with the long-term average (1953–2011) of 15.5°C during the same period. Meanwhile, the precipitation during the same period was only 115 mm in 2010 and 183 mm in 2011 with the long-term average of 177 mm. The growing season precipitation in 2010 was only about 65% of that in a normal year, confirming 2010 was a dry year (Fig. 2).

**Figure 3:**
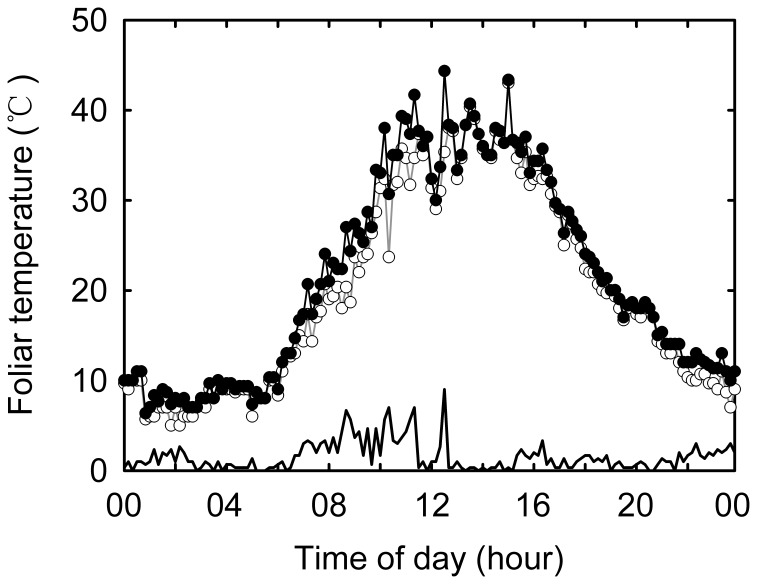
Representative 24-h foliar temperature (*T*_leaf_) profiles from *Stipa krylovii* Roshev. grown in the control (open) and warming (filled) plots during the field measurement campaigns. Thick solid line indicates warming-induced changes in *T*_leaf_ between control and warming plots.

The experimental warming significantly increased daytime *T*_leaf_ by 2.07°C (*P*<0.001), on average (Fig. 3). Warming increased daytime *T*_growth_ in the warming plots reaching 28.59 and 23.14°C in 2010 and 2011, respectively. Meanwhile, the daytime *T*_growth_ in the control plots was only 25.72 and 21.31°C in 2010 and 2011, respectively. The details of the warming effects on microclimate at the study site can be found in Wan *et al*. [44] and Xia *et al*. [45].

### Respiration

**Figure 4:**
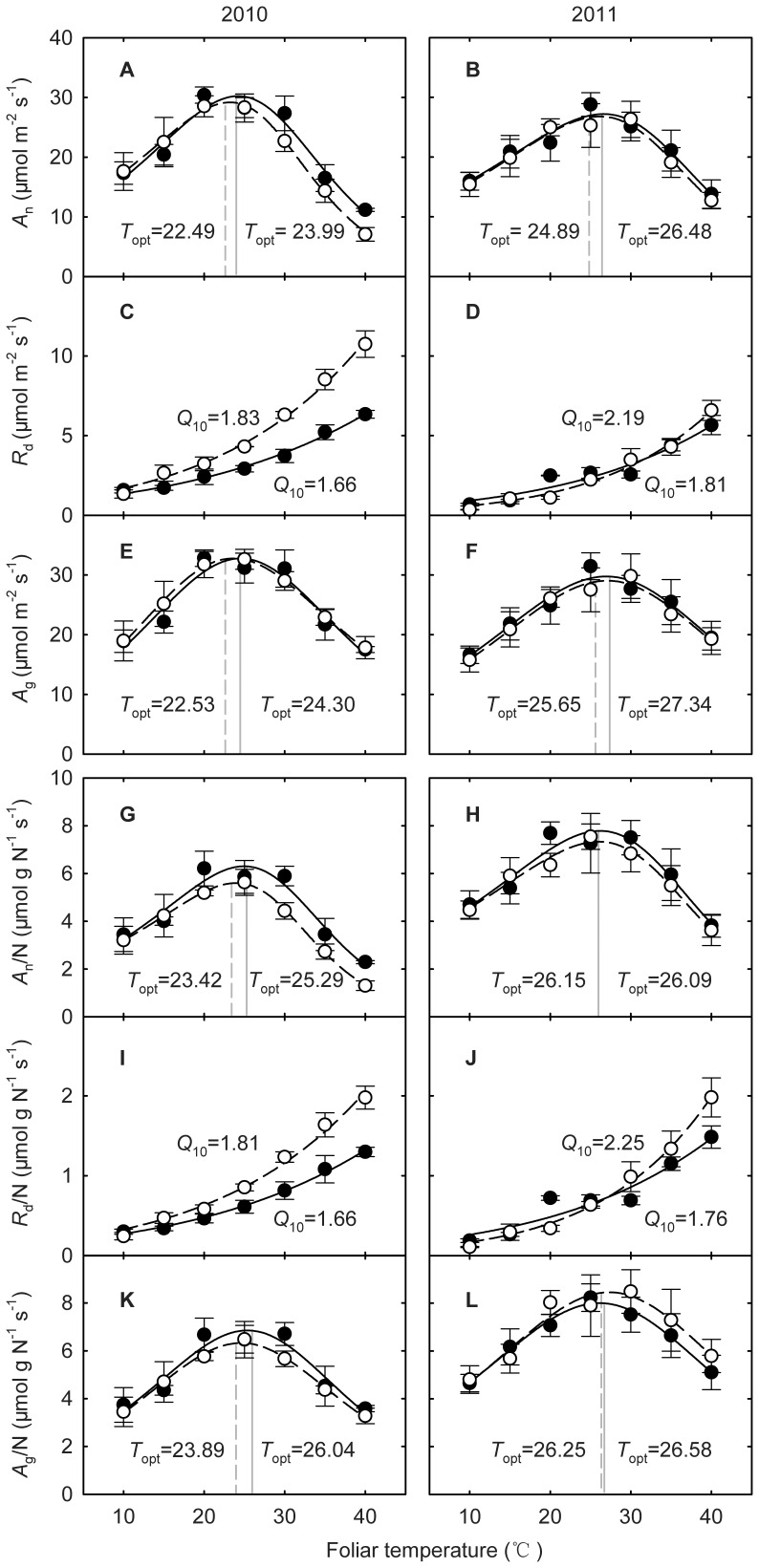
Warming effects on the responses of photosynthesis and respiration to foliar temperature (*T*_leaf_) in 2010 (left panels) and 2011 (right panels). The filled circles indicate the warming plots and the open circles for the control plots. (A) to (F) foliar area based: (A) and (B) net CO_2_ assimilation (*A*_n_); (C) and (D) dark respiration (*R*_d_); (E) and (F) gross CO_2_ assimilation (*A*_g_); (G) to (L) foliar nitrogen based: (G) and (H) *A*_n_ ; (I) and (J) *R*_d_; (K) and (L) *A*_g_. Each data point is the average of 4 replicates.

Warming significantly decreased respiratory temperature sensitivity, *Q*_10_, in both years (both *P*<0.05) (Fig. 4, Table 1). *Q*_10_ of *R*_d_ on a foliar area basis decreased from 1.83 in the control plots to 1.66 in the warming plots in 2010 (*P* = 0.049) (Table 2) and from 2.19 to 1.81 in 2011 (*P* = 0.042) (Table 3). Meanwhile, *Q*_10_ of *R*_d_ on a foliar N basis marginally decreased from 1.81 to 1.66 in 2010 (*P* = 0.094) and significantly decreased from 2.25 to 1.76 in 2011 (*P* = 0.011) (Table 2, 3). Warming marginally reduced base respiration rate at 10°C (*R*_10_) on a foliar area basis from 1.70 to 1.35 µmol m^−2^ s^−1^ in 2010 (*P* = 0.090) but increased that from 0.58 to 0.92 µmol m^−2^ s^−1^ in 2011 (*P* = 0.050) (Table 2, 3). Warming effects on the *R*_10_ on a foliar N basis were similar to the area-based *R*_d_ (Fig. 4).

**Table 1:** Results (*P*-values) of one-way ANOVA on the effects of warming on the responses of *A*_n_ (the net CO_2_ assimilation rate), *R*_d_ (dark respiration), *A*_g_ (the gross CO_2_ assimilation rate), *V*_cmax_ (the maximum rate of Rubisco carboxylation) and *J*_max_ (the maximum rate of photosynthetic electron transport) expressed per unit foliar area and nitrogen to instantaneous change (10–40°C within a 5 h period) in *T*_leaf_ (foliar temperature) in 2010 and 2011.

Year	Parameters	*c*	Δ*H*_a_	Δ*H*_d_	Δ*S*	*T*_opt_	*Q*_10_	*ref*_10_
2010	*A*_n_	0.836	0.844	0.735	0.727	0.310	/	0.816
	*R*_d_	**0.027**	**0.046**	/	/	/	**0.049**	0.090
	*A*_g_	0.292	0.300	0.979	0.913	0.328	/	0.839
	*V*_cmax_	0.055	0.064	/	/	/	0.062	0.784
	*J*_max_	0.879	0.842	0.757	0.772	0.520	/	0.181
	*A*_n_/N	0.726	0.732	0.575	0.612	0.323	/	0.955
	*R*_d_/N	0.071	0.095	/	/	/	0.094	0.094
	*A*_g_/N	0.977	0.976	0.138	0.178	0.302	/	0.996
	*V*_cmax_/N	0.150	0.158	/	/	/	0.142	0.546
	*J*_max_/N	0.474	0.468	0.678	0.646	0.874	/	0.381
2Δ011	*A*_n_	0.474	0.472	0.986	0.923	0.619	/	0.865
	*R*_d_	**0.042**	**0.040**	/	/	/	**0.042**	0.050
	*A*_g_	0.403	0.400	0.600	0.529	0.637	/	0.758
	*V*_cmax_	0.723	0.712	/	/	/	0.779	0.656
	*J*_max_	0.166	0.167	0.325	0.369	0.317	/	0.487
	*A*_n_/N	0.354	0.354	0.470	0.453	0.989	/	0.886
	*R*_d_/N	**0.010**	**0.010**	/	/	/	**0.011**	**0.026**
	*A*_g_/N	0.306	0.305	0.604	0.554	0.933	/	0.698
	*V*_cmax_/N	0.074	0.079	/	/	/	0.093	0.703
	*J*_max_/N	0.463	0.468	0.215	0.223	0.115	/	0.657

*c* is a scaling constant, Δ*H*_a_ is the activation energy, Δ*H*_d_ is a term for deactivation, Δ*S* is an entropy term, *T*_opt_ is the thermal optimum, *Q*_10_ is the temperature sensitivity and *ref*_10_ is the estimated basal rate at the reference temperature of 10°C. Significant values (*P*<0.05) are shown bold.

**Table 2:** Warming effects on the responses of *A*_n_ (the net CO_2_ assimilation rate), *R*_d_ (dark respiration), *A*_g_ (the gross CO_2_ assimilation rate), *V*_cmax_ (the maximum rate of Rubisco carboxylation) and *J*_max_ (the maximum rate of photosynthetic electron transport) expressed per unit foliar area and nitrogen to instantaneous change (10–40°C within a 5 h period) in *T*_leaf_ (foliar temperature) in the dry growing season (2010).

Parameters	Treatment	*c*	Δ*H*_a_	Δ*H*_d_	Δ*S*	*T*_opt_	*Q*_10_	*ref*_10_
*A*_n_ (µmol m^−2^ s^−1^)	Control	24.24±5.86	50.48±14.38	166.73±19.47	0.56±0.07	22.49±1.04	/	17.33±3.63
	Warming	25.73±3.69	53.96±8.92	158.17±14.21	0.53±0.04	23.99±0.87	/	16.34±1.87
*R*_d_ (µmol m^−2^ s^−1^)	Control	20.03±0.92	45.93±2.27	/	/	/	1.83±0.05	1.70±0.12
	Warming	16.59±0.75	38.37±1.97	/	/	/	1.66±0.04	1.35±0.13
*A*_g_ (µmol m^−2^ s^−1^)	Control	38.57±6.03	83.57±14.11	134.98±14.15	0.46±0.05	22.53±1.38	/	18.44±3.52
	Warming	29.77±4.66	63.18±11.13	135.46±9.63	0.45±0.03	24.30±0.91	/	17.59±1.88
*V*_cmax_ (µmol m^−2^ s^−1^)	Control	24.68±1.26	49.08±3.08	/	/	/	1.91±0.07	46.38±2.43
	Warming	21.64±0.25	41.97±0.62	/	/	/	1.74±0.01	45.33±2.73
*J*_max_ (µmol m^−2^ s^−1^)	Control	34.22±4.47	68.79±10.39	126.60±17.24	0.43±0.05	25.50±0.90	/	126.47±15.86
	Warming	35.24±4.58	71.89±10.69	132.40±4.80	0.44±0.01	26.55±1.24	/	101.15±5.31
*A*_n_/N (µmol g N^−1^ s^−1^)	Control	22.15±8.29	49.63±20.16	189.72±13.56	0.63±0.05	23.42±0.78	/	3.18±0.73
	Warming	27.95±13.45	63.21±32.05	201.66±14.95	0.67±0.06	25.29±1.55	/	3.13±0.58
*R*_d_/N (µmol g N^−1^ s^−1^)	Control	18.04±0.83	45.10±2.03	/	/	/	1.81±0.05	0.33±0.02
	Warming	15.06±1.08	38.51±2.64	/	/	/	1.66±0.06	0.28±0.02
*A*_g_/N (µmol g N^−1^ s^−1^)	Control	28.54±7.08	64.31±17.24	131.93±19.92	0.44±0.07	23.89±0.76	/	3.38±0.72
	Warming	29.01±13.69	65.48±32.55	176.38±16.66	0.58±0.06	26.04±1.75	/	3.39±0.60
*V*_cmax_/N (µmol g N^−1^ s^−1^)	Control	22.47±1.15	47.69±2.81	/	/	/	1.87±0.07	9.18±0.49
	Warming	19.79±1.15	41.26±2.83	/	/	/	1.72±0.06	9.68±0.61
*J*_max_/N (µmol g N^−1^ s^−1^)	Control	26.79±5.46	55.64±13.06	151.52±16.73	0.50±0.05	27.17±1.47	/	23.28±3.43
	Warming	35.83±10.49	77.39±24.84	165.48±27.26	0.55±0.09	27.58±1.98	/	19.04±2.88

*c* is a scaling constant, Δ*H*_a_ is the activation energy, Δ*H*_d_ is a term for deactivation, Δ*S* is an entropy term, *T*_opt_ is the thermal optimum, *Q*_10_ is the temperature sensitivity and *ref*_10_ is the estimated basal rate at the reference temperature of 10°C. Values are means (n = 4, ± SE).

**Table 3:** Warming effects on the responses of *A*_n_, *R*_d_, *A*_g_, *V*_cmax_ and *J*_max_ expressed per unit foliar area and nitrogen to instantaneous change (10–40°C within a 5 h period) in *T*_leaf_ in the wet growing season (2011).

Parameters	Treatment	*c*	Δ*H*_a_	Δ*H*_d_	Δ*S*	*T*_opt_	*Q*_10_	*ref*_10_
*A*_n_ (µmolm^−2^ s^−1^)	Control	30.91±9.71	66.17±22.71	175.99±18.90	0.59±0.06	24.89±2.47	/	15.07±2.49
	Warming	21.82±6.90	44.81±16.09	175.39±26.86	0.58±0.08	26.48±1.76	/	15.56±1.29
*R*_d_ (µmolm^−2^ s^−1^)	Control	24.93±1.23	60.02±3.13	/	/	/	2.19±0.09	0.58±0.09
	Warming	18.95±1.97	44.85±4.88	/	/	/	1.81±0.11	0.92±0.11
*A*_g_ (µmolm^−2^ s^−1^)	Control	39.19±13.54	85.44±31.59	167.09±24.98	0.56±0.08	25.65±2.77	/	15.27±2.45
	Warming	25.00±8.06	52.17±18.80	148.67±22.07	0.49±0.07	27.34±1.99	/	16.16±1.26
*V*_cmax_ (µmol m^−2^ s^−1^)	Control	23.98±0.80	47.78±2.07	/	/	/	1.87±0.05	40.65±4.82
	Warming	23.25±1.81	45.87±4.47	/	/	/	1.83±0.11	43.85±4.84
*J*_max_ (µmol m^−2^ s^−1^)	Control	27.81±5.52	54.61±13.31	167.70±27.42	0.54±0.08	31.56±1.01	/	102.85±15.77
	Warming	41.44±6.65	86.59±15.41	136.76±8.98	0.46±0.02	30.03±0.97	/	90.79±4.06
*A*_n_/N (µmol g N^−1^ s^−1^)	Control	28.91±9.48	64.42±22.35	277.95±128.81	0.91±0.41	26.15±3.34	/	4.38±0.71
	Warming	18.26±4.78	39.35±11.09	175.08±35.01	0.57±0.11	26.09±1.89	/	4.50±0.30
*R*_d_/N (µmol g N^−1^ s^−1^)	Control	24.49±1.52	62.01±3.83	/	/	/	2.25±0.11	0.16±0.03
	Warming	16.71±1.44	42.52±3.54	/	/	/	1.76±0.08	0.26±0.02
*A*_g_/N (µmol g N^−1^ s^−1^)	Control	37.55±11.17	84.51±26.22	193.11±55.95	0.64±0.17	26.25±3.03	/	4.37±0.64
	Warming	22.93±6.77	50.17±15.76	157.41±33.58	0.52±0.10	26.58±2.18	/	4.65±0.29
*V*_cmax_/N (µmol g N^−1^ s^−1^)	Control	23.24±0.69	48.97±1.84	/	/	/	1.90±0.05	11.67±1.37
	Warming	21.11±0.70	43.81±1.61	/	/	/	1.78±0.04	12.26±0.58
*J*_max_/N (µmol g N^−1^ s^−1^)	Control	27.40±9.59	56.67±22.90	218.93±62.97	0.71±0.20	32.46±1.55	/	28.09±3.70
	Warming	36.34±6.18	77.67±14.53	131.16±7.25	0.44±0.02	29.26±0.79	/	26.24±1.40

Values are means (n = 4, ± SE). See Table 2 for abbreviations defined.

### Photosynthesis

The *A*_n_/*T*_leaf_ curves were typically bell-shaped in both warming and control plots (Fig. 4). Warming had little effect on *T*_opt_ of *A*_n_ in both years (both *P*>0.05) (Table 1). *T*_opt_ of *A*_n_ on a foliar area basis was 22.49 and 23.99°C for the control and the warming plots respectively in 2010, and 24.89 and 26.48°C respectively in 2011 (Fig. 4). *T*_opt_ of *A*_g_ on a foliar area basis was 22.53 and 24.30°C for the control and the warming plots respectively in 2010 (*P* = 0.328), and 25.65 and 27.34°C respectively in 2011 (*P* = 0.637) (Fig. 4). Warming also had little effects on *T*_opt_ of *A*_n_ and *A*_g_ on a foliar N basis in either 2010 or 2011 (all *P*>0.05) (Table 1).

### Biochemical Limitations to Photosynthesis

**Figure 5:**
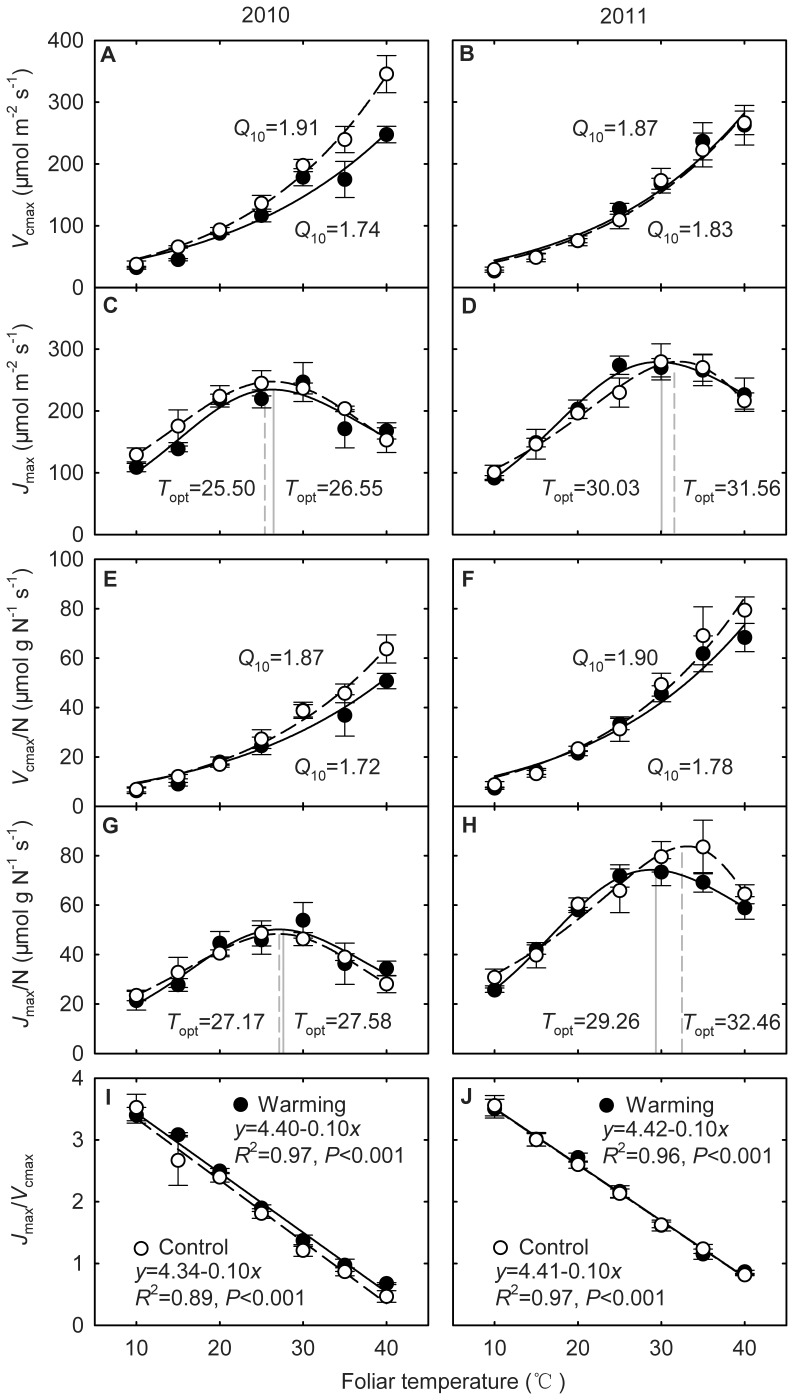
Warming effects on the responses of the maximum rate of Rubisco carboxylation (*V*_cmax_), the maximum rate of photosynthetic electron transport (*J*_max_) and the *J*_max_/*V*_cmax_ ratio to foliar temperature (*T*_leaf_) in 2010 (left panels) and 2011 (right panels). The filled circles indicate the warming plots and the open circles for the control plots. (A) and (B) area-based *V*_cmax_; (C) and (D) area-based *J*_max_; (E) and (F) N-based *V*_cmax_; (G) and (H) N-based *J*_max_; (I) and (J) the *J*_max_/*V*_cmax_ ratio. Each data point is the average of 4 replicates.

The effects of warming on *Q*_10_ of *V*_cmax_ were not statistically significant between the warming and the control plots in both years (both *P*>0.05) (Table 1), but we found a general decreasing trend from the control to warming plots (Fig. 5). *Q*_10_ of *V*_cmax_ on a foliar area basis was 1.91 and 1.74 for the control and the warming plots respectively in 2010 (*P* = 0.062), and 1.87 and 1.83 respectively in 2011 (*P* = 0.779) (Fig. 5, Table 2, 3). *Q*_10_ of *V*_cmax_ on a foliar N basis was 1.87 and 1.72 for the control and the warming plots respectively in 2010 (*P* = 0.174), and 1.90 and 1.78 respectively in 2011 (*P* = 0.668) (Fig. 5, Table 2, 3). The warming effects on *Q*_10_ of *J*_max_ were not be detected in 2010 or 2011 (both *P*>0.05) (Table 1). In addition, the warming effects on the slope and *y*-intercept of the temperature-response curves for *J*_max_/*V*_cmax_ ratio were not statistically significant (all *P*>0.05), though the ratio decreased linearly with the *T*_leaf_ (Fig. 5).

### Diffusion Limitations to Photosynthesis

**Figure 6:**
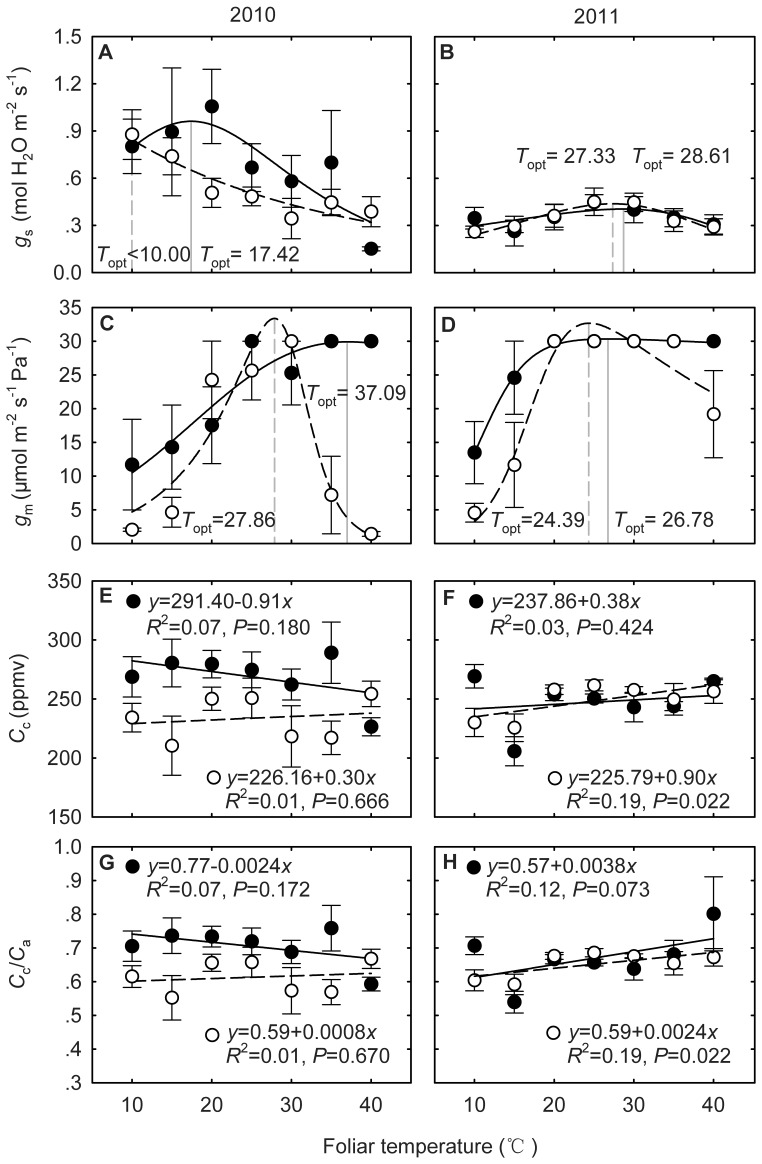
Warming effects on the responses of stomatal conductance (*g*_s_) (A, B), mesophyll conductance (*g*_m_) (C, D), carboxylation site CO_2_ concentrations (*C*_c_) (E, F), and *C*_c_/*C*_a_ ratio (G, H) to foliar temperature (*T*_leaf_) in 2010 (left panels) and 2011 (right panels). The filled circles indicate the warming plots and the open circles for the control plots. Each data point is the average of 4 replicates. **Note:** *g*_m_ is constrained to be 30 (µmol m^−2^ s^−1^ Pa^−1^) or less.

In 2010, a dry year, *g*_s_ in the warming plots was marginally greater than that in the control plots (*P* = 0.137), and *T*_opt_ for *g*_s_ was about 17.42°C in the warming plots and less than 10°C in the control plots (Fig. 6). The *g*_m_ in the warming plots was significantly greater than that in the control plots (*P*<0.001), and *T*_opt_ for *g*_m_ appeared at 37.09°C in the warming plots and 27.86°C in the control plots (Fig. 6). *C*_c_ in the warming plots was approximately 35 ppmv greater than that in the control plots (*P*<0.001), but *C*_c_ was independent of *T*_leaf_ in both the warming and the control plots (both *P*>0.05) (Fig. 6). Similarly, *C*_c_/*C*_a_ ratio was constant and independent of *T*_leaf_ in the warming and the control plots (both *P*>0.05) (Fig. 6). However, experimental warming significantly increased *C*_c_/*C*_a_ ratio in 2010 (*P* = 0.001) with an average value of 0.70 in the warming plots and 0.61 in the control plots (Fig. 6).

In 2011, a wet year, Warming had little effect on *g*_s_ and *g*_m_ (both *P*>0.05), which resulted in no difference in *C*_c_ between the warming and the control plots (*P* = 0.860) (Fig. 6). Experimental warming also had little effect on *C*_c_/*C*_a_ ratio in 2011 (*P* = 0.447) with an average value of 0.67 in the warming plots and 0.65 in the control plots (Fig. 6).

### Foliar Characteristics

Warming marginally decreased foliar N concentration in 2010 (*P* = 0.063), but significantly increased that in 2011 (*P* = 0.002) (Table 4). Warming had little effect on foliar carbon concentration in both years (both *P*>0.05). Foliar C/N ratio was significantly higher in the warming plots than in the control plots in 2010 (*P*<0.001) and the opposite was true in 2011 (Table 4).

**Table 4:** Foliar characteristics of *Stipa krylovii* Roshev. grown in the control and warming plots.

Year	Treatment	N concentration	C concentration	C/N ratio
2010	Control	5.34±0.07	86.66±1.48	16.22±0.16
	Warming	5.02±0.16	92.91±3.42	18.48±0.26
	*P* value	0.063	0.100	**<0.001**
2011	Control	3.41±0.05	78.12±1.35	22.92±0.15
	Warming	3.68±0.06	79.70±1.44	21.65±0.11
	*P* value	**0.002**	0.426	**<0.001**

Warming effects on foliar nitrogen concentrations (g N m^−2^), carbon concentrations (g C m^−2^) and C/N ratio (g g^−1^) were analyzed using one-way ANOVA for each year. Significant values (*P*<0.05) are shown bold (Mean ± SE, N = 28).

## Discussion

### Acclimation of Respiration

**Figure 7:**
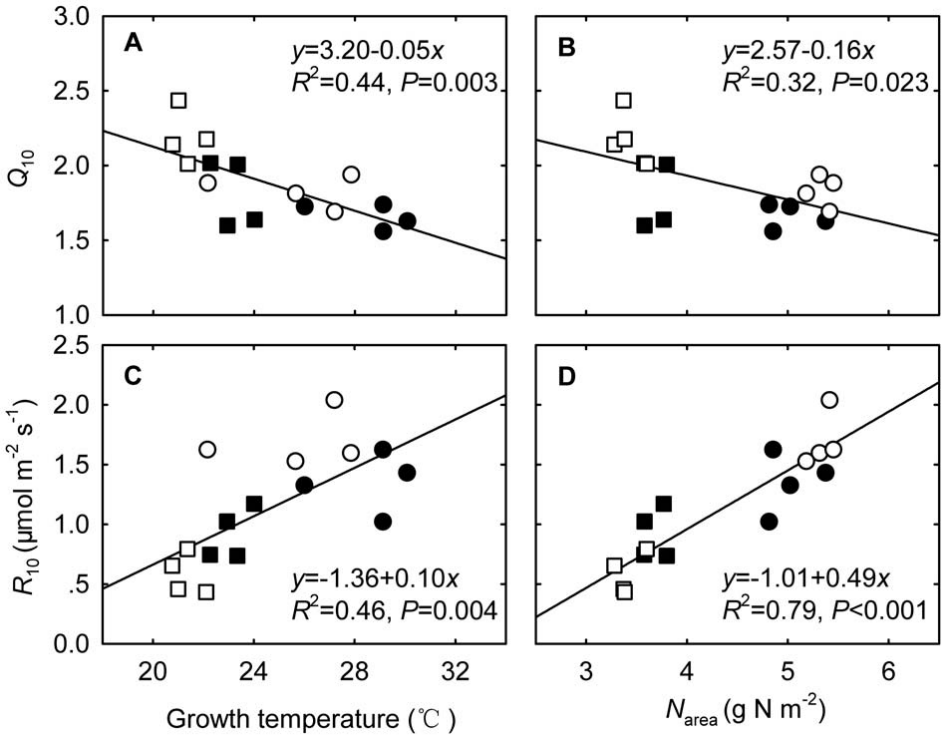
Responses of *Q*_10_ (the temperature sensitivity) (top panel) and *R*_10_ (the estimated basal respiration rate at the reference temperature of 10°C) (lower panel) in the control (open) and warming (filled) plots in 2010 (circles) and 2011 (squares) to *T*_growth_ (left panel) and foliar nitrogen concentrations (right panel), respectively. Values are means (n = 4, ± SE).

*R*_d_ was sensitive to *T*_leaf_ with the *R*_d_/*T*_leaf_ relationship following a typical exponential curve, but warming reduced the magnitude (Fig. 4, Table S1). Our results are consistent with previous studies [18], [20], [62] that the temperature sensitivity of *R*_d_ is negatively related to the *T*_growth_ (Fig. 7). According to the respiratory acclimation mechanisms proposed by Atkin and Tjoelker [11], the temperature-mediated change in *Q*_10_ is determined by the maximum enzyme activity and/or substrate availability [1], [17], [20]. Earlier results from the same warming experiment confirmed that day warming significantly reduced foliar starch concentrations (–6.1%, *P* = 0.009), suggesting the reduction in *Q*_10_ in the current study might be attributed to the lower substrate concentrations.

Foliar N concentrations induced by experimental warming in our study may also affect the temperature sensitivity of *R*_d_, *Q*_10_ (Fig. 7). To date, few studies have examined the role of N in the change in *Q*_10_. Turnbull *et al*. [63] found that *Q*_10_ of *R*_d_ for the trees in a temperate rainforest increased with increasing N availability along a soil chronosequence in New Zealand. However, Ow *et al*. [64] have reported that N had little or no impact on *Q*_10_ of *R*_d_ when saplings grown at high and low N availabilities were transferred to a different *T*_growth_ regime. Here, we found a negative correlation between *Q*_10_ of *R*_d_ and foliar N concentrations (Fig. 7). The detailed mechanisms are not clear, but the confounding effect of foliar N concentrations with other factors, such as temperature and precipitation, may have played an important role in the “apparent” *Q*_10_ [11], [65], [66].

In the current study we found that experimental warming marginally reduced base respiration rate at 10°C (*R*_10_) in 2010 but increased that in 2011 (Table 2, 3). This could have been attributed to the differential responses of foliar N concentration to warming in the two hydrologically contrasting growing seasons. Warming marginally decreased foliar N concentration in the dry growing season (2010), but increased that in the wet growing season (2011) (Table 4). A growing number of studies [8], [14], [17], including our current study, have found that foliar N concentration was strongly related to *R*_10_ (Fig. 7). Therefore, we believed that foliar N concentration played an important role in the diverging responses of *R*_10_ to warming in both years.

### Acclimation of Photosynthesis

Photosynthesis has long been known to acclimate to prevailing *T*_growth_ by shifting the *T*_opt_ [67]. For example, Gunderson *et al*. [60] have reported that a 3-year warming of 2–4°C has resulted in a higher *T*_opt_ of *A*_n_ for five species of deciduous trees. In the current study we found that a 6-year warming of 2.07°C did not resulted in changes in *T*_opt_ of *A*_n_ (Fig. 4, Table S1). We also found that there were not statistically significant differences between the shift in *T*_opt_ of *A*_n_ and *A*_g_ in 2010 (*P* = 0.896) or 2011 (*P* = 0.984). This suggests that the instantaneous response of photosynthesis was independent of changes in *R*_d_.

It has been proposed that the increase in the temperature sensitivity of *V*_cmax_, indicated by Δ*H*_a_ of *V*_cmax_, contributed to the thermal acclimation of photosynthesis to experimental warming [36], [61], [68]. However, in the current study we found that warming slightly decreased Δ*H*_a_ of *V*_cmax_ (Fig. 5, Table 1). Biochemically, the change in Δ*H*_a_ of *V*_cmax_ is closely related to the temperature dependence of Rubisco activity [69], Rubisco activation status [70], [71], dimorphism of Rubisco [31], and the amount of Rubisco [72]. The lower Δ*H*_a_ of *V*_cmax_ obtained from the warming plots indicated that warming slightly decreased the temperature sensitivity of those processes.

**Figure 8:**
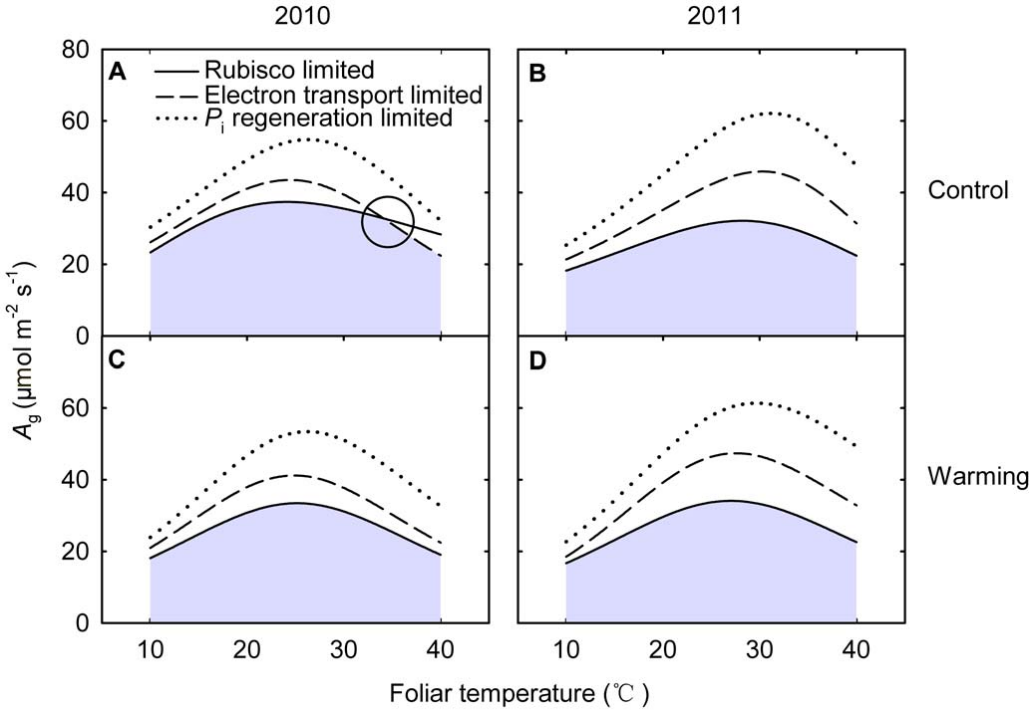
Warming effects on the responses of biochemical limitations in gross CO_2_ assimilation (*A*_g_) to foliar temperature (*T*_leaf_) at chloroplast partial pressure of CO_2_ (*C*_c_) of 250.8 ppmv in 2010 (left panels) and 2011 (right panels). The top panels indicate the control plots and the lower panels for the warming plots. *C*_c_ was set at 250.8 ppmv considering that the mean *C*_c_/*C*_a_ ratio was 0.66 at ambient CO_2_ concentration (380 ppmv) for all the *A*_n_/*C*_i_ curves measured. The response of *A*_g_ is delineated by the minimum value of either Rubisco-limited (solid curve), ribulose bisphosphate (RuBP) regeneration-limited (dashed curve) and *P*_i_ regeneration-limited (dotted curve). Circle indicates co-limited point, moving from the Rubisco-limited state to RuBP regeneration-limited state.

Previous studies found that RuBP regeneration processes may play an important role in the thermal acclimation of photosynthesis [34], [39], [73]. The increase in the thermal stability of photosystem II, indicated by Δ*H*_a_ of *J*_max_, has been shown to be related to the thermal acclimation of *A*_g_ to warming [34]–[36], [74]. However, in the current study we found only minor response of Δ*H*_a_ of *J*_max_ to warming (Fig. 5, Table 1). This is also confirmed by our results that the RuBP regeneration seldom limited *A*_g_ (Fig. 8).

A number of studies have reported that the balance between the carboxylation and the regeneration of RuBP, indicated by *J*_max_/*V*_cmax_ ratio, can also affect the thermal acclimation of photosynthesis [39], [75]. In our study, the experimental warming had little effect on the linear trend of *J*_max_/*V*_cmax_ ratio to *T*_leaf_ (Fig. 5). Nevertheless, in this study we found that *J*_max_/*V*_cmax_ ratio declined sharply and linearly with the instantaneous increase in *T*_leaf_ (Fig. 5). Many ecosystem models, such as Biome-BGC [76], have set *J*_max_/*V*_cmax_ ratio as a constant (2.1) which is independent of *T*_leaf_. Wullschleger [77] analyzed 164 *A*_n_/*C*_i_ curves for 109 C_3_ plant species which were measured under *T*_leaf_ ranging from 13 to 35°C and found the average *J*_max_/*V*_cmax_ ratio was 2.1. Others found that *J*_max_/*V*_cmax_ ratio was not a constant instead varying with *T*_leaf_ through a linear [51], [78]–[80] or nonlinear relationship [81]. Our current results show that the relationship (between *J*_max_ and *V*_cmax_) itself is highly temperature dependent, suggesting that photosynthesis models have to consider the temperature dependence of *J*_max_/*V*_cmax_ ratio.

In addition to biochemical limitations, the thermal acclimation of photosynthesis may also relate to CO_2_ diffusion processes in leaves and chloroplasts, such as *g*_s_ and *g*_m_, because changes in *T*_growth_ may affect CO_2_ diffusivity, solubility, membrane permeability and stomatal movement [82]–[85]. Previous studies have found that increasing *g*_s_ and/or *g*_m_ can cause the increase of *T*_opt_ of *A*_n_ [36], [40], [41], [67], [86]. In the current study we found that warming increased *g*_m_ (Fig. 6) in 2010 which might contribute to the modest variation in *T*_opt_ of *A*_g_ in 2010. However, we found smaller increases in *g*_s_ and *g*_m_ (Fig. 6) in 2011, which may explain the weaker acclimation in 2011 (Fig. 4). The differential responses of CO_2_ diffusion process to warming in the two hydrologically contrasting growing seasons could have been attributed to changes in soil moisture and N availability induced by warming [87]. It is noted that, so far, no consistent conclusions have been achieved on the warming effect on *g*_s_ and *g*_m_. Some researchers found that warming increased *g*_s_ [39], [88]–[90] and *g*_m_ [91], and others found warming decreased *g*_s_ [92] and *g*_m_ [61], or no effect on *g*_s_ [93] and *g*_m_ [40]. Those various studies suggest that other factors, such as warming-induced water depletion and change in N availability, may have interacting effects on responses of CO_2_ diffusion process to warming. These results call for multi-factor experiments, such as the combination of warming with water manipulation and fertilization [21], for understanding the mechanisms of thermal acclimation of photosynthesis under future global change.

### Balance between Respiration and Photosynthesis

**Figure 9:**
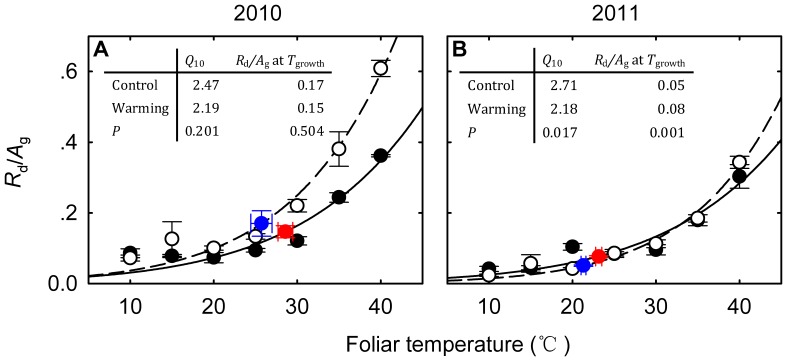
Warming effects on the response of *R*_d_/*A*_g_ ratio (balance between dark respiration and gross CO_2_ assimilation) to instantaneous change (10–40°C within a 5 h period) in *T*_leaf_ (foliar temperature) in the dry growing season (2010) (A) and the wet growing season (2011) (B). The filled circles indicate the warming plots and the open circles for the control plots. The blue and red circles indicate *R*_d_/*A*_g_ ratio at growth temperature (*T*_growth_), computed using the thermal dynamic properties (individual Δ*H*_a_ and *c* values for each plot) and the *T*_growth_.

The acclimation of foliar respiration and photosynthesis is also reflected in *R*/*A* ratio which indicates the balance between carbon gain, loss and accumulation [1], [2]. Our results show that the instantaneous (<5 h) warming at foliage level has non-linearly increased *R*_d_/*A*_g_ ratio, indicating proportionally more carbon loss through *R*_d_ as *T*_leaf_ goes up (Fig. 9). However, the 6-year experimental warming has resulted in thermal acclimation of the grasses as evidenced by the decrease of the curvature of the response curve of *R*_d_/*A*_g_ ratio to *T*_leaf_ (Fig. 9). It is important to note that though the balance between *R*_d_ and *A*_g_ was re-established through the thermal acclimation [6], [8], [9], [18], *R*_d_/*A*_g_ ratio was still increasing with *T*_growth_ in a wet year (Fig. 9). This means that, at foliage level, acclimation can only partially compensate the negative impact from the global warming.

## Supporting Information

Table S1 **Results (** ***P*** **-values) of two-way ANOVA on the effects of warming, year, and both interactions on the responses of** ***A*** **_n_ (the net CO_2_ assimilation rate),** ***R*** **_d_ (dark respiration),** ***A*** **_g_ (the gross CO_2_ assimilation rate),** ***V*** **_cmax_ (the maximum rate of Rubisco carboxylation) and** ***J*** **_max_ (the maximum rate of photosynthetic electron transport) expressed per unit foliar area and nitrogen to instantaneous change (10–40°C within a 5 h period) in** ***T*** **_leaf_ (foliar temperature).** *c* is a scaling constant, Δ*H*_a_ is the activation energy, Δ*H*_d_ is a term for deactivation, Δ*S* is an entropy term, *T*_opt_ is the thermal optimum, *Q*_10_ is the temperature sensitivity and *ref*_10_ is the estimated basal rate at the reference temperature of 10°C. Significant values (*P*<0.05) are shown bold.
(DOC)

Appendix S1 **User’s guide for the** ***A*** **/** ***C*** **_c_ curve fitting model with measured respiration, modified based on Sharkey** ***et al*** **.’s** [**50**] **Microsoft Excel spreadsheet-based software to reduce the number of fitting parameters (** ***R*** **_d_ is fixed in the model), version 1.2 (Last updated 25 July, 2012).**
(XLS)
