# Supplementary material for: Acclimation of Foliar Respiration and Photosynthesis in Response to Experimental Warming in a Temperate Steppe in Northern China
Source: PLoS One. 2013 Feb 15;8(2):e56482. doi: 10.1371/journal.pone.0056482 (PMC3574119; doi:10.1371/journal.pone.0056482)
Supplement: Table S1 — Results ( P -values) of two-way ANOVA on the effects of warming, year, and both interactions on the responses of A n (the net CO2 assimilation rate), R d (dark respiration), A g (the gross CO2 assimilation rate), V cmax (the maximum rate of Rubisco carboxylation) and J max (the maximum rate of photosynthetic electron transport) expressed per unit foliar area and nitrogen to instantaneous change (10–40°C within a 5 h period) in T leaf (foliar temperature). c is a scaling constant, ΔH a is the activation energy, ΔH d is a term for deactivation, ΔS is an entropy term, T opt is the thermal optimum, Q 10 is the temperature sensitivity and ref 10 is the estimated basal rate at the reference temperature of 10°C. Significant values (P<0.05) are shown bold. (DOC) [file pone.0056482.s001.doc]

**Support Information**

**Table S1** Results (*P*-values) of two-way ANOVA on the effects of warming, year, and both interactions on the responses of *A*_n_ (the net CO_2_ assimilation rate), *R*_d_ (dark respiration), *A*_g_ (the gross CO_2_ assimilation rate), *V*_cmax_ (the maximum rate of Rubisco carboxylation) and *J*_max_ (the maximum rate of photosynthetic electron transport) expressed per unit foliar area and nitrogen to instantaneous change (10-40℃ within a 5 h period) in *T*_leaf_ (foliar temperature). *c* is a scaling constant, Δ*H*_a_ is the activation energy, Δ*H*_d_ is a term for deactivation, Δ*S* is an entropy term, *T*_opt_ is the thermal optimum, *Q*_10_ is the temperature sensitivity and *ref*_10_ is the estimated basal rate at the reference temperature of 10 ℃. Significant values (*P*< 0.05) are shown bold.

|  | Parameters | *c* | Δ*H*_a_ | Δ*H*_d_ | Δ*S* | *T*_opt_ | *Q*_10_ | *ref*_10_ |
| --- | --- | --- | --- | --- | --- | --- | --- | --- |
| Warming | *A*_n_ | 0.591 | 0.593 | 0.826 | 0.765 | 0.371 | / | 0.922 |
|  | *R*_d_ | **0.003** | **0.005** | / | / | / | **0.006** | 0.961 |
|  | *A*_g_ | 0.214 | 0.214 | 0.641 | 0.531 | 0.380 | / | 0.994 |
|  | *V*_cmax_ | 0.135 | 0.149 | / | / | / | 0.167 | 0.785 |
|  | *J*_max_ | 0.198 | 0.190 | 0.473 | 0.524 | 0.820 | / | 0.135 |
|  | *A*_n_/N | 0.803 | 0.804 | 0.513 | 0.498 | 0.674 | / | 0.961 |
|  | *R*_d_/N | **0.001** | **0.001** | / | / | / | **0.001** | 0.328 |
|  | *A*_g_/N | 0.407 | 0.410 | 0.149 | 0.189 | 0.294 | / | 0.808 |
|  | *V*_cmax_/N | **0.026** | **0.029** | / | / | / | **0.027** | 0.527 |
|  | *J*_max_/N | 0.295 | 0.295 | 0.319 | 0.347 | 0.372 | / | 0.329 |
| Year | *A*_n_ | 0.845 | 0.844 | 0.528 | 0.551 | 0.167 | / | 0.551 |
|  | *R*_d_ | **0.016** | **0.008** | / | / | / | **0.007** | **0.001** |
|  | *A*_g_ | 0.817 | 0.827 | 0.250 | 0.286 | 0.130 | / | 0.361 |
|  | *V*_cmax_ | 0.708 | 0.666 | / | / | / | 0.691 | 0.370 |
|  | *J*_max_ | 0.985 | 0.984 | 0.205 | 0.226 | **0.001** | / | 0.171 |
|  | *A*_n_/N | 0.881 | 0.845 | 0.656 | 0.667 | 0.420 | / | 0.053 |
|  | *R*_d_/N | **0.007** | **0.005** | / | / | / | **0.005** | **0.001** |
|  | *A*_g_/N | 0.787 | 0.819 | 0.679 | 0.700 | 0.695 | / | 0.080 |
|  | *V*_cmax_/N | 0.293 | 0.429 | / | / | / | 0.489 | **0.011** |
|  | *J*_max_/N | 0.947 | 0.974 | 0.650 | 0.674 | **0.039** | / | 0.068 |
| Warming×Year | *A*_n_ | 0.457 | 0.460 | 0.848 | 0.885 | 0.979 | / | 0.769 |
|  | *R*_d_ | 0.350 | 0.267 | / | / | / | 0.221 | 0.890 |
|  | *A*_g_ | 0.764 | 0.758 | 0.623 | 0.600 | 0.985 | / | 0.727 |
|  | *V*_cmax_ | 0.347 | 0.391 | / | / | / | 0.349 | 0.593 |
|  | *J*_max_ | 0.264 | 0.275 | 0.300 | 0.346 | 0.239 | / | 0.580 |
|  | *A*_n_/N | 0.404 | 0.411 | 0.412 | 0.395 | 0.656 | / | 0.890 |
|  | *R*_d_/N | 0.078 | 0.059 | / | / | / | **0.044** | **0.004** |
|  | *A*_g_/N | 0.381 | 0.383 | 0.689 | 0.638 | 0.923 | / | 0.814 |
|  | *V*_cmax_/N | 0.777 | 0.792 | / | / | / | 0.770 | 0.957 |
|  | *J*_max_/N | 0.996 | 0.985 | 0.177 | 0.179 | 0.255 | / | 0.696 |
